# Extracts from Correspondence

**Published:** 1859-06

**Authors:** 


### EXTRACTS FROM CORRESPONDENCE.

Searcy's Hook.—Dr. Reuben Searcy, of Tuscaloosa, Ala., writes—I have invented a little hook for the purpose of extracting seeds, grains, berries, and pebbles from the nose and ears of children, that succeeds to my entire satisfaction. It is quickly and easily made wherever a pin can be procured. Bend a pin's point at right angles scarcely the twentieth of an inch, or about half a line, cut the head of the pin off, split a little stick, insert a third of the pin, and secure it with a waxed thread. You can grapple with the point of this instrument, the berry, grain or seed, and extract it easily. It is also the best instrument for extracting pebbles—by guiding its point sideways beyond the pebble, turning the point so it will catch it, and pressing the pin slightly against the pebble, and drawing out the instrument, you will either slide the pebble out or you will give it one revolution outward; this repeated, will soon put you in possession of the pebble.

